# Supplementary material for: Architecting Nanostructured Co-BTC@GO Composites for Supercapacitor Electrode Application
Source: Nanomaterials (Basel). 2022 Sep 18;12(18):3234. doi: 10.3390/nano12183234 (PMC9505437; doi:10.3390/nano12183234)
Supplement: Supplementary file 1 [file nanomaterials-12-03234-s001.zip › nanomaterials-1919095-supplementary.pdf]

Supplementary Materials

Architecting Nanostructured Co-BTC@GO Composites for Supercapacitor Electrode Application

Tianen Chen [1], Allen Yang [2], Wei Zhang [3], Jinhui Nie [1], Tingting Wang [1], Jianchao Gong [1], Yuanhao Wang [1,*] and Yaxiong Ji [1,*]

1 Hoffmann Institute of Advanced Materials, Shenzhen Polytechnic, Shenzhen 518055, China
2 Nord Anglia International School, Hong Kong, China
3 PetroChina Petrochemical Research Institute, Beijing 102206, China
* Correspondence: wangyuanhao@szpt.edu.cn (Y.W.); kobejyx@126.com (Y.J.); Tel.: +86-15818696045 (Y.J.)

Electrochemical Analysis

All electrochemical measurements were performed by using an electrochemical working station (CHI660E, Chenhua, Shanghai, China) apparatus in a three-electrode system. The measurements were carried out in 3M aqueous KOH electrolyte. The working electrodes were prepared in an agate mortar by mixing the active composite, carbon black, and PVDF in a mass ratio of 80:10:10 in NMP as a solvent. The current collector was prepared by dispersing 2.5 mg of mixture onto nickel foam (1 cm × 1cm), where the mass of the active ingredient was 2 mg. An Ag/AgCl electrode was used as the reference electrode and Pt wire (1 cm × 1 cm) was used as the counter electrode. Cyclic voltammograms (CV) were acquired at 0–0.6 V at various scan rates. Galvanostatic charge-discharge (GCD) curves were obtained in the potential range of 0–0.5 V at the different current densities. The electrochemical impedance spectroscopy (EIS) of the mixture was acquired in the frequency range of 1×10^5 Hz to 0.01 Hz with an AC potential amplitude of 0.005 V at 0.325 V DC voltage [1–4].

The specific capacitance is calculated based on the GCD curves by using Equation (S1) as following [5]:

$$C = \frac{I \times \Delta t}{m \times \Delta V} \tag{S1}$$

in which C (F/g) is the specific capacity, I (A) is the discharge current, Δt (s) is the discharge time, m (g) is the mass of active materials and ΔV (V) is the potential range of discharge, respectively.

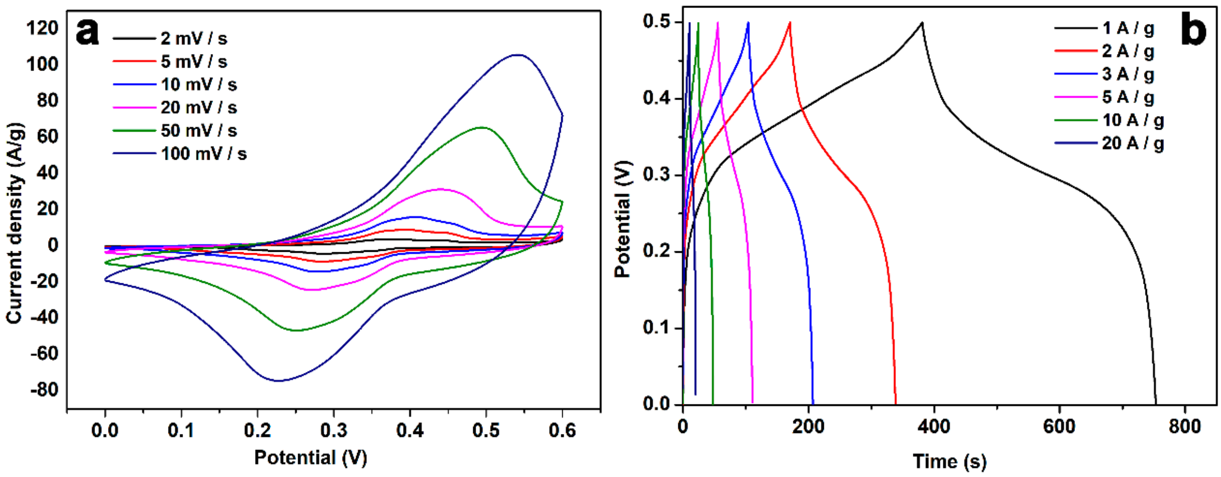

Figure S1. (**a**) CV curves of Co-BTC at different scan rates, (**b**) GCD curves of Co-BTC at different current densities.

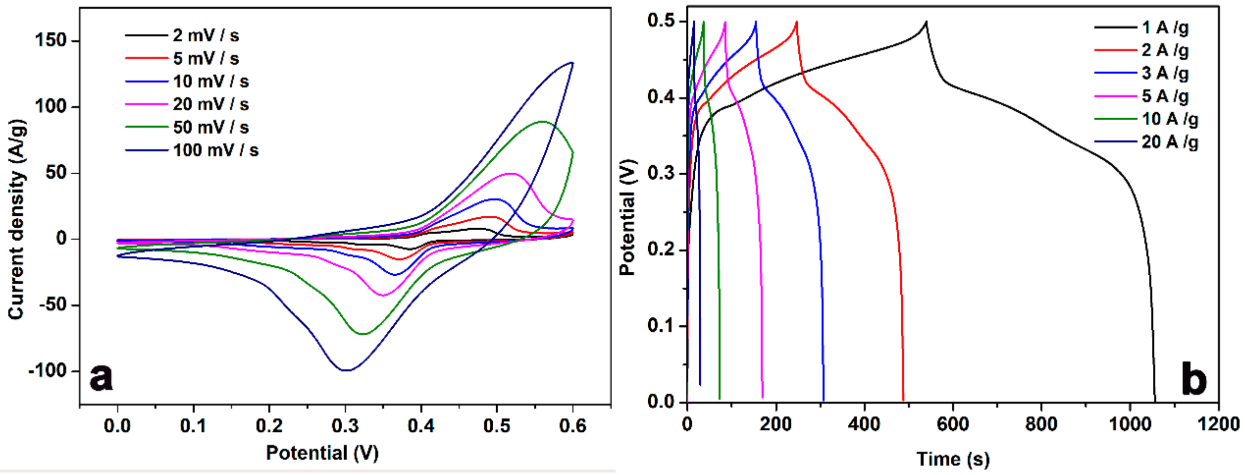

Figure S2. (a) CV curves of Co-BTC@GO 1 at different scan rates, (b) GCD curves of Co-BTC@GO 1 at different current densities.

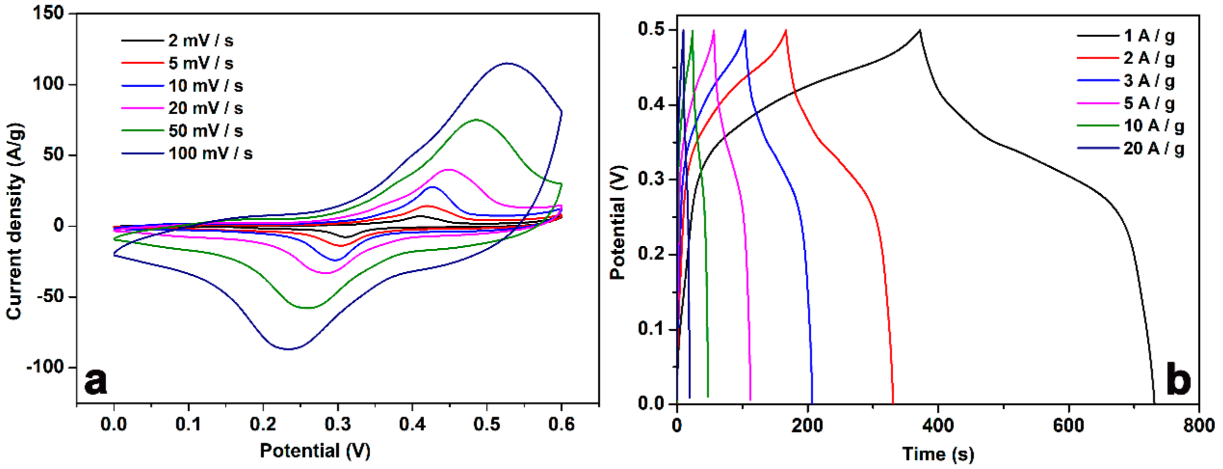

Figure S3. (a) CV curves of Co-BTC@GO 4 at different scan rates, (b) GCD curves of Co-BTC@GO 4 at different current densities.

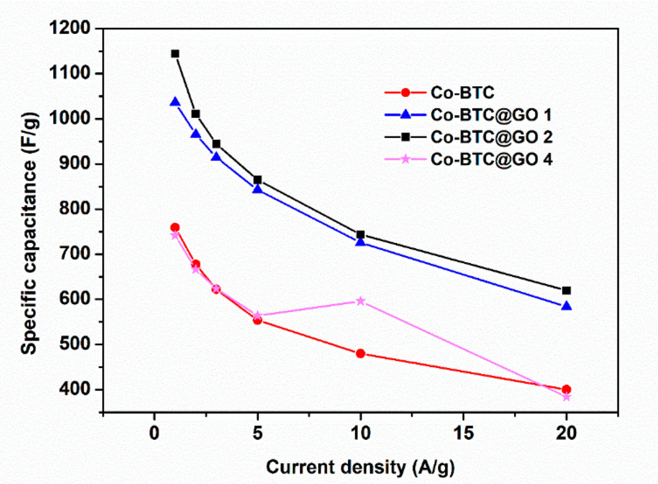

Figure S4. specific capacitance at various current densities (1–20 A/g) of Co-BTC, Co-BTC@GO 1, Co-BTC@GO 2 and Co-BTC@GO 4.

Reference

1. Zhang, H.; Sun, Y.; Zhang, X.; Yang, H.; Lin, B. A New Straightforward Uncalcined Approach for Morphology Modulating to Enhance the Electrical Capacity Performance of Co-MOF. *Electrochim. Acta* **2021**, *389*, 138684. https://doi.org/10.1016/j.electacta.2021.138684

2. Cong, H.P.; Ren, X.C.; Wang, P.; Yu, S.H. Flexible Graphene-Polyaniline Composite Paper for High-Performance Supercapacitor. *Energy Environ. Sci.* **2013**, *6*, 1185–1191. https://doi.org/10.1039/c2ee24203f.
3. Li, B.; Cao, H.; Yin, G.; Lu, Y.; Yin, J. Cu_2O@reduced Graphene Oxide Composite for Removal of Contaminants from Water and Supercapacitors. *J. Mater. Chem.* **2011**, *21*, 10645–10648. https://doi.org/10.1039/c1jm12135a
4. Yang, J.; Yu, C.; Fan, X.; Ling, Z.; Qiu, J.; Gogotsi, Y. Facile Fabrication of MWCNT-Doped NiCoAl-Layered Double Hydroxide Nanosheets with Enhanced Electrochemical Performances. *J. Mater. Chem. A* **2013**, *1*, 1963–1968. https://doi.org/10.1039/c2ta00832g.
5. Zhang, Y.; Lin, B.; Sun, Y.; Han, P.; Wang, J.; Ding, X.; Zhang, X.; Yang, H. MoO_2@Cu@C Composites Prepared by Using Polyoxometalates@Metal-Organic Frameworks as Template for All-Solid-State Flexible Supercapacitor. *Electrochim. Acta* **2016**, *188*, 490–498. https://doi.org/10.1016/j.electacta.2015.12.037.
